# Biochemical and Clinical Features of Insulinoma in a Patient with Turner Syndrome

**DOI:** 10.1155/2019/6809479

**Published:** 2019-02-10

**Authors:** Darius A. Schneider, M. Zare, F. Behnia, M. Matesan, T. Tylee

**Affiliations:** ^1^University of Washington, Department of Medicine, Seattle, WA, USA; ^2^Department of Radiology, Harborview Medical Center, Seattle, WA, USA

## Abstract

Turner syndrome (TS), i.e., mosaic or nonmosaic states with only one normal X chromosome in females, is characterized by a wide spectrum of somatic, hormonal, and metabolic features. Here we report an unusual case of recurrent hypoglycemia in a 53-year-old woman with TS. Biochemical work-up following a 72h fast revealed detectable, inappropriate for low glucose insulin levels and elevated proinsulin and beta-hydroxybutyrate (BOHB) levels. MR and multiphase CT showed a solid 2.5 cm pancreatic tail mass with absent uptake in the ^111^In-pentetreotide (Octreoscan) scan. Subsequent hepatic vein blood sampling after intra-arterial calcium stimulation showed sharp increase in insulin and modest increase in proinsulin levels. The patient underwent excision of the mass with resolution of symptoms. Histopathologic examination confirmed the neuroendocrine etiology of the tumor. This is, to our knowledge, the third report of TS and concomitant insulinoma. Impaired counterregulatory response to hypoglycemia in patients with TS may result in symptomatic hypoglycemia with only mild insulin elevation and elevated proinsulin in setting of hypoglycemia may be the only indication of insulinoma in these patients. BOHB levels should not be used for ruling out EHH in patients with TS.

## 1. Introduction

TS is a chromosomal abnormality, affecting one in 2,000 female live-births [1]. X-chromosome monosomy (45XO karyotype) accounts for >55% of Turner syndrome cases, while mosaicism, mainly 45XO/46XX or 45XO/47XXX, has been detected in the remaining cases.

Endocrinopathies associated with TS include growth hormone resistance, insulin resistance, and diabetes [1].

Insulinomas are neuroendocrine tumors arising from the pancreatic beta-cells and most common cause of endogenous hyperinsulinemic hypoglycemia (EHH) [2]. Their incidence is 2-4 cases per million individuals per year [2]. Prominent features are fasting hypoglycemia with elevated serum insulin and proinsulin levels. Recently, two cases of histologically confirmed insulinoma were described in women with TS [3, 4]. Here we present a third case of a pancreatic tail insulinoma in a patient with TS with interesting clinical/biochemical features: severe, symptomatic hypoglycemia despite rather low, yet inappropriately detectable insulin levels following 72h fast; sharp increase of insulin levels upon arterial calcium stimulation; and elevated BOHB levels.

## 2. Case Report

A 53-year-old woman with small stature and dysmorphic features consistent with TS, not previously diagnosed with diabetes, presented to the endocrinology clinic for evaluation of episodes of hypoglycemia <20mg/dl with transient loss of consciousness. She was admitted to the inpatient service and underwent a controlled 72h fast. After 64h, she developed hypoglycemia to the low 30s mg/dl with mild neuroglycopenic symptoms (emotional outbursts). Biochemical workup showed unsuppressed insulin levels (1.2mIU/L), markedly elevated proinsulin (30 pmol/L), and BOHB levels (6500*μ*mol/L).

**Figure 1:**
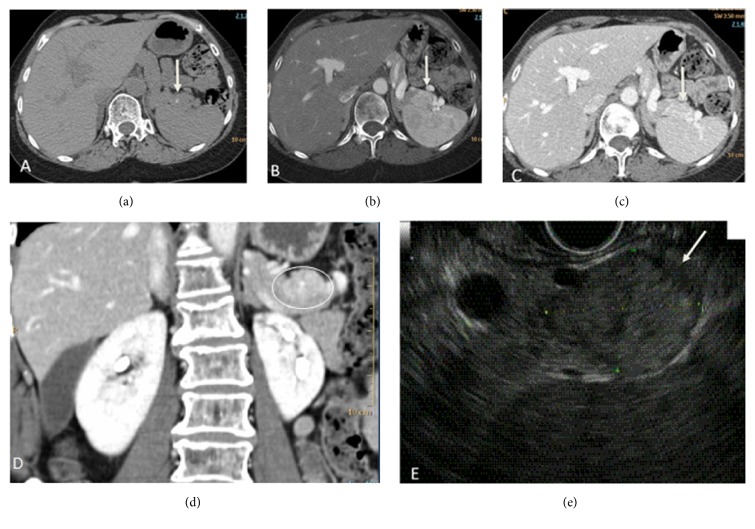
Axial noncontrast CT (a) shows a small calcification at the tail of the pancreas. Axial arterial phase CT (b) shows a 2.5 cm hyperenhancing pancreatic mass which on venous phase ((c) and (d)) shows slightly more heterogeneity compared to the spleen. Endoscopic ultrasound (e) showed heterogeneous hypoechoic 1.7 x 2.5 cm pancreatic tail mass with calcifications.

**Figure 2:**
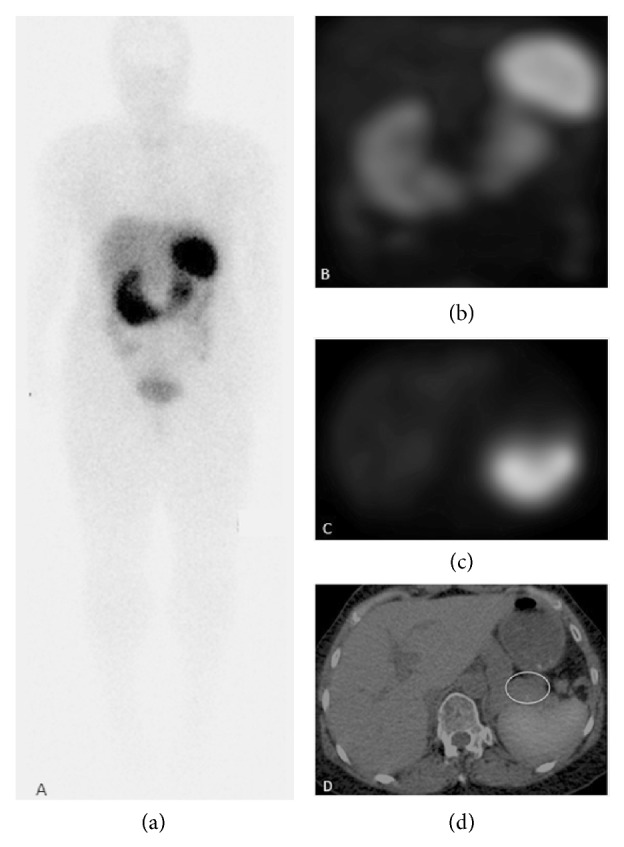
24-hour ^111^In-pentetreotide scan. Anterior whole body planar image (a), MIP reconstruction of SPECT acquisition (b), axial SPECT image (c), and fused axial SPECT/CT image (d) show normal biodistribution of the radiotracer and no uptake in the pancreatic tail mass, ruling out a splenule.

MR and multiphase CT (Figure 1) showed a solid, hyperenhancing 2.5cm pancreatic tail mass with slow washout. ^111^In-pentetreotide (Octreoscan) study (Figure 2) demonstrated absence of radiotracer uptake ruling out a splenule. CT guided fine needle biopsy of the mass confirmed neuroendocrine etiology. Since Octreoscan sensitivity is not high, hepatic vein blood sampling after intra-arterial calcium stimulation was further performed and showed robust increase of insulin (9.4 to 17.8mIU/L) and proinsulin (7pmol/L to 20pmol/L) and high c-peptide levels at the distal splenic artery, consistent with insulinoma of the tail of the pancreas.

Surgical excision of the mass resulted in complete resolution of symptoms up to 3 years postop. Histologic examination confirmed a well differentiated neuroendocrine tumor with immunostainings positive for chromogranin and synaptophysin and a KI index of <2%.

## 3. Discussion

Insulinomas are rare neuroendocrine tumors with estimated incidence of 1 case per 250,000 patient-years [2]. They are more common in women and usually occur in the fifth or sixth decade [2]. The tumor should be suspected in a patient presenting with spontaneous fasting hypoglycemia and represent the most prevalent cause of EHH [2].

Although a slightly increased incidence of solid tumors has been described in patients with TS, insulinoma prevalence is not generally thought to be increased in women with TS [5]. Thus far, there were two cases reported of insulinoma in a patient with 45X TS and in a patient with mosaic 45XO/47XXX TS [3, 4].

Here, we present a case of an insulinoma in a 53-year-old woman with TS and reoccurring episodes of severe, life-threatening hypoglycemia. A 72h fast test was positive, with hypoglycemia below 30mg/dl and mild neuroglycopenic symptoms. Biochemical analysis revealed low yet inappropriately detectable insulin and c-peptide levels, and high proinsulin levels. Beta-hydroxybutyrate (BHOB) levels were markedly elevated. Arterial calcium stimulation test confirmed both hyperinsulinemia and hyperproinsulinemia in the distal splenic artery, localizing the mass in the pancreatic tail, consistent with imaging studies. Surgical removal of the pancreatic mass led to complete and consistent resolution of symptoms.

Some aspects of this case were somewhat unusual and, hence, worth discussing: (i) hypoglycemia occurred relatively late during the 72h fast in our patient (normally after <48h of fasting for insulinomas [2]). This may be caused by underlying insulin resistance and/or increased hepatic glucose output in patients with TS and has not been described before; (ii) despite severe fasting hypoglycaemia with neuroglycopenic symptoms and clearly positive 72h fast test, measured insulin levels were only modest compared to previously described insulinoma cases [6], albeit inappropriately elevated relative to low glucose. This may be the result of an impaired hormonal (epinephrine, growth hormone, and glucagon) counterregulatory response to hypoglycemia in people with TS. While this exact mechanism has not been previously described in TS, it has been shown that catecholamine release [7] is diminished in adults with TS. Hence, low yet detectable insulin levels in TS patients with documented hypoglycemia should prompt evaluation for insulinoma, as impaired counterregulatory hormones may result in symptomatic hypoglycemia with only mild insulin elevation.

While insulin levels were only modestly increased, proinsulin levels in the 72h fast test were robustly elevated, consistent with previous literature [6]. Both insulin and proinsulin levels increased sharply upon arterial calcium stimulation. The discrepancy between insulin and proinsulin levels may be explained by insulin resistance and/or impaired beta-cellular processing of proinsulin to insulin in this patient with TS. Elevated proinsulin in the setting of hypoglycemia may be the only indication of insulinoma for TS patients.

Furthermore, we saw markedly elevated BOHB levels. This may seem surprising, since elevated BOHB levels are thought to rule out EHH with 100% sensitivity and specificity [8]; however, as in this case and consistent with previously described cases [9] their diagnostic specificity is much lower when plasma glucose levels drop below 45mg/dl. High BOHB levels may be explained by the combination of modest elevation in insulin levels and increased insulin resistance specific for TS, facilitating the escape of ketogenesis from the inhibitory effects of insulin, as previously described in setting of posthypoglycemia insulin resistance [10]. Hence, BOHB levels should not be used for ruling out EHH in patients with TS.

## 4. Conclusion

The development of insulinoma in patients with Turner syndrome has to be considered if there is clinical suspicion for hyperinsulinemic hypoglycemia. Due to impairments of insulin sensitivity and glycemic counterregulation inherent to patients with TS, symptomatic hypoglycemia may result with only mild insulin elevation and beta-hydroxybutyrate (BOHB) levels may be elevated and should not be used for ruling out EHH. Elevated proinsulin in the setting of hypoglycemia may be the only indication of insulinoma.

This is, thus far, the third case of TS presenting with insulinoma; considering the rarity of both TS and insulinoma, a potential underlying association may be worth exploring.
